# A case series of osseous metastases in patients with glioblastoma

**DOI:** 10.1097/MD.0000000000038794

**Published:** 2024-07-05

**Authors:** Lauren Michelle Webb, Mason J. Webb, Jian L. Campian, Samantha J. Caron, Michael W. Ruff, Joon H. Uhm, Ugur Sener

**Affiliations:** a Department of Neurology, Mayo Clinic, Rochester, Minnesota, USA; b Department of Hematology/Oncology, Mayo Clinic, Rochester, Minnesota, USA; c Department of Medical Oncology, Mayo Clinic, Rochester, Minnesota, USA.

**Keywords:** glioblastoma, metastases, osseous

## Abstract

**Background:**

Extracranial metastases occur in <2% of cases of glioblastoma (GBM). When metastases do occur, bone is the most common destination. Herein, we review clinical characteristics of GBM patients with osseous metastases and evaluate both potential risk factors and prognostic significance.

**Methods:**

Using an institutional database, we identified and retrospectively analyzed 6 patients with both GBM and osseous metastases. We collected data on patient demographics, tumor genetics, clinical courses, and outcomes. Given the rarity of metastatic GBM, we conducted historical comparisons using previously published literature.

**Results:**

Five patients with osseous metastases (83%) were male, with a median age of 46 years at GBM diagnosis (range: 20–84). All patients had *IDH*-wildtype, *MGMT* promoter unmethylated GBM and 5 (83%) had alterations in *TP53*. All patients underwent surgical resection for GBM followed by radiation with concurrent and adjuvant temozolomide. Four patients (67%) received bevacizumab prior to bone metastasis diagnosis. Bone metastases were discovered at a median of 12.2 months (range: 5.3–35.2) after GBM diagnosis and 4.8 months after starting bevacizumab (range: 3.5–13.2). Three patients (50%) received immunotherapy. After osseous metastasis diagnosis, the median survival was 25 days (range: 13–225).

**Conclusion:**

In our cohort, most patients were male and young at the time of GBM diagnosis. All patients had *IDH*-wildtype, *MGMT* promoter unmethylated GBM, and most had alterations in *TP53*, which may be important for osseous metastasis. Most patients received bevacizumab, which has been associated with earlier metastasis. Osseous metastases of GBM occur and portend a dismal prognosis in an already aggressive malignancy.

## 1. Introduction

Glioblastoma (GBM) is the most common primary brain tumor in adults, comprising 49% of primary malignant central nervous system (CNS) tumors.^[1]^ Treatment of GBM involves maximal safe surgical resection followed by radiation with concomitant and adjuvant temozolomide, with or without tumor-treating fields.^[2]^ Despite multimodal treatment, tumor recurrence is universal and median survival is poor at 15 months.^[3]^

Despite the aggressive nature of GBM, extracranial metastasis is rare, occurring in <2% of cases.^[4]^ The rarity of extracranial metastasis from GBM has been attributed to multiple factors, including containment by the blood-brain barrier, lack of traditional lymphatic channels in the CNS, control of extracranial GBM by the immune system, and the challenge of GBM flourishing in extracellular matrices outside of the brain.^[5]^ The window of opportunity for extracranial metastasis is also temporally limited due to GBM poor prognosis, as most reported cases of metastasis occur in the setting of advanced intracranial disease.^[6]^ Nonetheless, GBM is the most common primary CNS tumor to metastasize.^[7]^

Multiple mechanisms of GBM spread have been proposed. Hematogenous dissemination is a major suspected route of metastasis. Circulating tumor cells have been discovered in the blood of 21% of patients with GBM and may lead to future metastasis.^[5]^ GBM metastases have been theorized to start by gaining access to the bloodstream via tumor-mediated intracranial angiogenesis or because of surgical resections which disrupt the blood-brain barrier. However, this hypothesis is either incorrect or incomplete, as GBM extracranial metastases have occurred in patients who have never had surgery.^[5]^ Furthermore, circulating tumor cells are not significantly increased after surgery, as would be expected if surgery-induced metastatic seeding is an etiology.^[5]^

Propagation of GBM through the cerebrospinal fluid is another possibility, especially for patients with ventriculoperitoneal shunts^[4]^ and metastasis within the CNS. Other routes for extracranial invasion by GBM cells could include the glial glymphatic system, direct invasion of the skull, and transneuronal spread along cranial and peripheral nerves.^[4,6]^

Risk factors for extracranial metastasis of GBM include male sex, surgical opening of the ventricles, and immunocompromised state.^[8]^ Gliosarcomas, accounting for 2% of all patients diagnosed with GBM, have more frequently been reported to metastasize extracranially.^[9]^ Age <60 years old is also associated with GBM metastasis, which may be related to longer survival increasing the risk of metastasis.^[4]^ The most common sites of GBM metastases include bone, lymph nodes, the lungs, and the liver.^[4]^

GBM bony metastases may be lytic or sclerotic and 63% involve the vertebral column. Other common locations include the skull, sternum, ribs, and appendicular skeleton.^[10]^ The rich venous plexus surrounding vertebral bodies may facilitate hematogenous spread to the spinal column.^[10]^ Similar to other spinal metastases, the most common region for GBM metastasis is the thoracic vertebrae.^[10]^

In this single-center case series, we review clinical characteristics of GBM patients with osseous metastases and evaluate potential risk factors and prognostic significance. We also compare our findings to the published literature.

## 2. Methods

We retrospectively analyzed the clinical presentations, diagnostic results, treatments, and clinical outcomes of 6 patients with GBM with osseous metastases at Mayo Clinic. The study was approved by the Mayo Clinic Institutional Review Board. This is a retrospective case series with no experimental intervention performed as part of the study. As such, informed consent was not obtained from the included patients, in accordance with regulations of the Institutional Review Board at Mayo Clinic.

We used an institutional research tool to identify patients. Our inclusion criteria were: pathologic diagnosis of GBM; diagnostic imaging confirming osseous metastasis; either pathologic confirmation of GBM as the etiology of the osseous metastasis or lack of a known alternative malignancy to explain the metastasis. Patients with radiographic evidence of osseous metastases who had a known secondary malignancy in addition to GBM and did not have histologic confirmation for the underlying etiology of the presumed osseous metastases were excluded.

Collected demographic parameters included: sex, age at GBM diagnosis, and ethnicity. The clinical details we collected included initial treatments for GBM (surgery, radiation, chemotherapy, tumor-treating fields), second- and third-line therapies and their timing after diagnosis, symptoms attributed to osseous metastases, presence of lymphopenia or hypercalcemia, timing of osseous metastasis discovery, osseous metastasis location and type (sclerotic or lytic), presence of metastases to other organs, and patient overall survival. Genetic data from patients’ intracranial GBMs were available and recorded. We examined the available imaging of osseous metastases which included magnetic resonance imaging, positron emission tomography, and computed tomography scans.

### 2.1. Statistical analysis

We report data as medians (range, minimum-maximum) for continuous variables and as frequencies and percentages for categorical variables.

## 3. Results

### 3.1. Patient demographics

Of the 6 patients we identified with osseous metastases of GBM, 5 (83%) were male, with a median age of 46 years (range, 20–84) at initial GBM diagnosis (Table 1). All patients were Caucasian.

**Table 1:** Clinical characteristics, treatments, and outcomes of patients with osseous metastases of GBM.

	Age at GBM diagnosis (yr)	Sex	Intracranial GBM location	Mutational status	Surgery	Concurrent TMZ/RT with adjuvant TMZ	Salvage therapy or immunotherapy	Bev	Duration of bev (mo)	Osseous metastasis locations	Extracranial metastasis to death (d)	Total survival (mo)
Patient 1	20	M	Anterior parasagittal region, both frontal lobes	• *IDH*-WT • *MGMT* UM • *TP53* overexpression	STR	Yes	None	Yes	4.1	C and T vertebrae	30	6.4
Patient 2	69	M	R inferior temporal lobe and R cingulate gyrus	• *IDH*-WT • *MGMT* UM • *TP53* underexpression	STR	Yes	None	No	N/A	Humerus, rib, thoracic and S vertebrae	13	6.1
Patient 3	41	M	L temporal pole	• *IDH*-WT • *MGMT* UM • *TERT* mutation • *PTEN* mutation • *TP53* missense mutation (loss of function)	GTR × 3	Yes	Lomustine Regorafenib* Allogeneic tumor lysate-pulsed autologous dendritic cell vaccination†	Yes*	N/A	Greater wing of the sphenoid	225	21.1
Patient 4	51	F	L frontotemporal lobe	• *IDH*-WT • *MGMT* UM • *TERT* mutation • *TP53* underexpression	STR	Yes	Lomustine	Yes	3.5	Skull	27	13.3
Patient5	37	M	L temporal lobe	• *IDH*-WT • *MGMT* UM • *EGFR* amplification • *TERT* mutation • *PTEN* mutation • Gain of chromosome 7 • Loss of chromosome 10	GTR	Yes	Lomustine Repeat radiation (35 Gy) Retifanlimab and epacadostat‡	Yes	5	T and lumbar vertebrae	19	27.6
Patient 6	84	M	L temporal lobe	• *IDH*-WT • *MGMT* UM • *TERT* mutation • *TP53* mutation	STR	Yes—proton beam RT (40 Gy)§	Pembrolizumab	Yes	13.4	Skull, ribs, pelvis, sternum, proximal long bones, all vertebrae	22	36.8

Bev = bevacizumab; C = cervical; F = female; GBM = glioblastoma; GTR = gross total resection; L = left; M = male; R = right; RT = radiotherapy; S = sacral; STR = subtotal resection; T = thoracic; TMZ = temozolomide; UM = promoter unmethylated; WT = wildtype.

* After osseous metastasis was discovered.

† Clinical trial: NCT03360708.

‡ Clinical trial: NCT03532295.

§ Clinical trial: NCT03778294.

### 3.2. GBM pathology

All patients had GBM, *IDH*-wildtype. *MGMT* promoter methylation was absent in all cases. Five of 6 patients (83%) had alterations in *TP53*. Two patients (33%) had gliosarcoma morphology. One patient (patient 2) had multifocal intracranial GBM. GBM involved the left temporal lobe in 4 patients (67%). Additional details of GBM location and genetics are presented in Table 1.

### 3.3. Patient clinical courses

**Figure 1.**
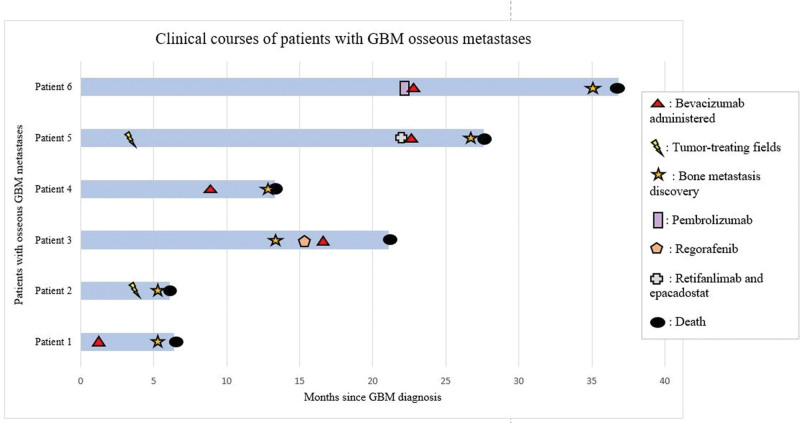
Swimmer plot depicting the clinical courses of the 6 patients, including major treatment events, bone metastasis discovery, and death.

All patients underwent surgical resection of their tumors. Gross total resection was achieved in 4 cases (67%), while 2 patients (33%) had subtotal resection. All patients received radiation therapy with concurrent and adjuvant temozolomide. One patient (patient 6) received 40 Gy proton beam radiation in 10 fractions as part of a clinical trial (NCT03778294). The remaining patients received 60 Gy in 30 fractions. Two patients (33%) also received tumor-treating fields. Additional GBM treatments are listed in Table 1. Anti-vascular endothelial growth factor agent, bevacizumab, was utilized in 5 of 6 patients (83%), for treatment-related vasogenic edema (n = 4, 66%) and for tumor progression (n = 2, 33%). Two patients (33%) underwent repeat surgical resections for recurrent intracranial GBM and 1 patient (17%) received repeat radiation. Additional chemotherapies included lomustine (n = 3, 50%), regorafenib (n = 1, 17%), a combination of retifanlimab and epacadostat as part of a clinical trial (n = 1, 17%) (NCT03532295), and pembrolizumab (n = 1, 17%). One patient (17%) enrolled in a clinical trial (NCT03360708) and received allogeneic tumor lysate-pulsed autologous dendritic cell vaccination. Patients’ clinical courses are displayed in Figure 1.

### 3.4. Clinical presentation of GBM with osseous metastasis

**Figure 2.**
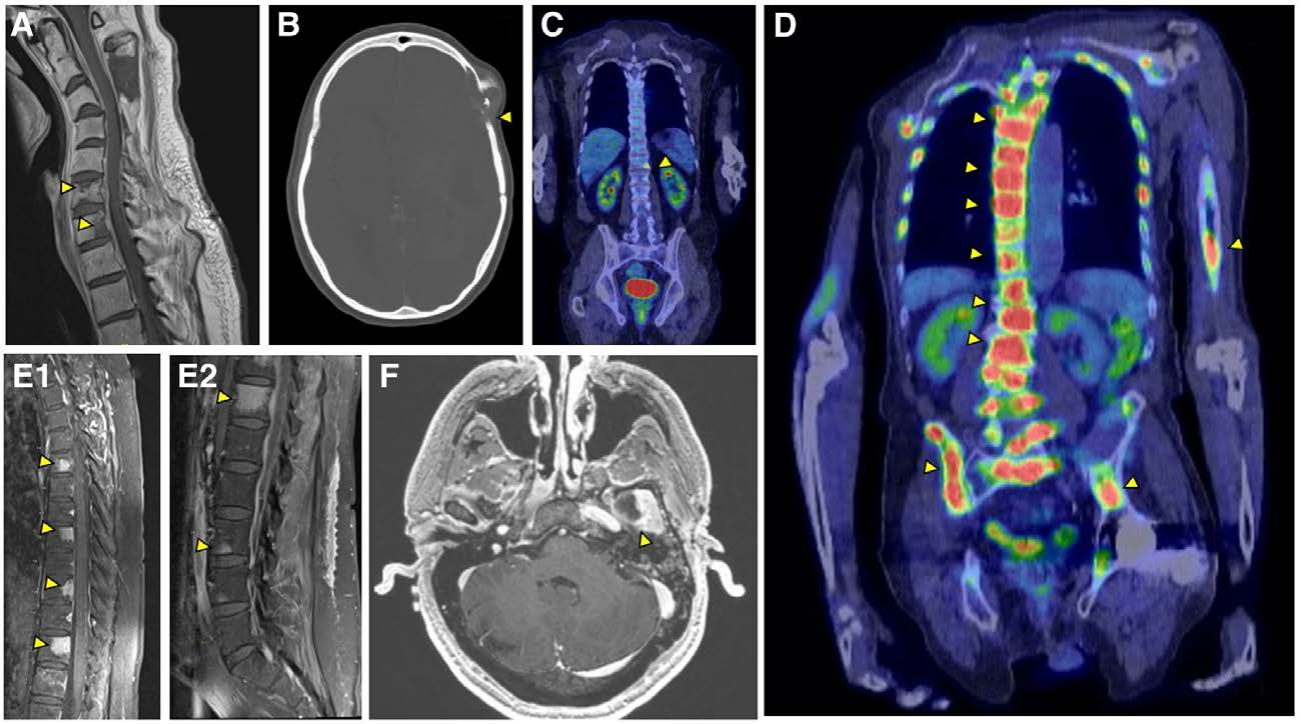
(A) T1-weighted, gadolinium-enhanced cervical spinal cord MRI demonstrating cervical vertebral metastases (Patient 1). (B) Head CT scan without intravenous contrast displaying lytic changes in the left frontotemporal calvarium (Patient 4). (C) PET-CT scan showing multiple thoracic vertebral metastases (Patient 2). (D) PET-CT scan demonstrating right proximal humerus and diffuse vertebral and pelvic osseous metastases (Patient 6). (E1–2) T1-weighted, gadolinium-enhanced MRI of the thoracic and lumbar spine showing scattered vertebral metastases (Patient 5). (F) Gadolinium-enhanced brain MRI revealing an osseous lesion involving the greater wing of the left sphenoid bone (Patient 3). CT = computed tomography; MRI = magnetic resonance imaging; PET = positron emission tomography.

Two of 6 patients (33%) experienced symptoms referable to their bone metastases with 1 patient experiencing back pain, headache, and leg weakness, and another experiencing urinary incontinence and gait disturbance. The other 4 patients (67%) had incidental discovery of their osseous metastases by imaging obtained for unrelated indications. Osseous metastases were detected by magnetic resonance imaging of the spine (n = 2) and brain (n = 1), positron emission tomography scan of the body (n = 2), and computed tomography scan of the head (n = 1) (Fig. 2).

Bone metastases of GBM were pathologically confirmed by biopsy in 3 patients (50%). The other 3 patients had no other known malignancy to explain their metastases. Three patients (50%) had metastases to other organs, including the spinal cord epidural space (n = 1), parotid gland (n = 1), lymph nodes (n = 2), liver (n = 1), adrenal gland (n = 1), and colon (n = 1).

All patients were lymphopenic (median lymphocyte count nadir: 0.73 × 10^9^/L, range: 0.41–2 × 10^9^/L) at the time of osseous metastasis discovery. None of the patients had hypercalcemia during their treatment course for GBM or after diagnosis of osseous metastases.

Osseous metastases were found a median of 13 months from initial GBM diagnosis (range, 5.4–35.7 months). For the 4 patients who received bevacizumab prior to osseous metastasis discovery, the median duration between initiation of bevacizumab and osseous metastasis discovery was 4.5 months (range, 1.3–22.4 months).

### 3.5. Patient outcomes

The median overall survival from initial diagnosis of GBM was 17.2 months among our patients. Patients died a median of 24.5 days (range: 13–225) after discovery of osseous metastases of GBM on imaging.

### 3.6. Reported cases of GBM osseous metastases in the literature

Numerous cases in the literature of GBM with osseous metastases have been reported in the literature,^[10]^ many of which include molecular data and treatment with bevacizumab and/or immunotherapies (Table 2). For the 18 total cases with tumor molecular data available, *MGMT* promoter methylation was absent in 15 (83%). Nine of fifteen patients (60%) were treated with bevacizumab. In this larger group of patients with osseous metastases of GBM, the median duration between extracranial metastasis to death was 3 months. Overall survival after GBM diagnosis was 27.6 months among the 21 patients with available information.

**Table 2:** Reported cases in literature of extracranial metastasis of GBM with available mutational data.

Publication	Age at GBM diagnosis (yr)	Sex	*IDH* status	*MGMT* promoter methylation status	Treatment with immunotherapy	Treatment with bev	Extracranial metastasis to death (mo)	Overall survival (mo)
Forsyth et al^[11]^	59	F	WT	−	No	Yes	3.5	9.5
Khattab et al^[12]^	51	M	Unknown	−	No	Yes	6	46
Xu et al^[13]^	58	F	WT	−	No	No	25	54
Simonetti et al^[14]^	38	M	WT	−	No	Yes	2	45
Ricard et al^[15]^	37	M	WT	Unknown	Pembrolizumab	Yes	2	139
Nagata et al^[16]^	46	F	WT	Unknown	No	No	1	6
Umphlett et al^[17]^	74	F	WT	−	No	No	4	12
Colamaria et al^[18]^	46	F	Unknown	+	No	No	Unknown	Unknown
den Hartog et al,^[19]^ case 7	63	M	WT	−	Unknown	Unknown	3	7
den Hartog et al,^[19]^ case 11	59	M	WT	Unknown	Unknown	Unknown	Unknown	Unknown
den Hartog et al,^[19]^ case 15	33	M	Mutant	Unknown	Unknown	Unknown	23	85
den Hartog et al,^[19]^ case 21	42	M	WT	+	Unknown	Unknown	24	29
den Hartog et al,^[19]^ case 24	55	F	WT	Unknown	Unknown	Unknown	0	7
Noch et al,^[20]^ case 2	39	M	WT	+	Unknown	Unknown	1	12.1
Noch et al,^[20]^ case 6	28	M	Mutant	−	Unknown	Unknown	5	37.5
Noch et al,^[20]^ case 7	23	F	Unknown	+	Unknown	Unknown	16.1	57.5
Zhang et al,^[21]^ case 13	47	M	WT	−	No	No	12	43
Present case, patient 1	20	M	WT	−	No	Yes	1	6.4
Present case, patient 2	69	M	WT	−	No	No	0.4	6.1
Present case, patient 3	41	M	WT	−	Regorafenib*	Yes*	7.5	21.1
Present case, patient 4	51	F	WT	−	No	Yes	0.9	13.3
Present case, patient 5	37	M	WT	−	Retifanlimab and epacadostat	Yes	0.6	27.6
Present case, patient 6	84	M	WT	−	Pembrolizumab	Yes	0.7	36.8
Summary	Median age: 46 (range 20–84)	15/24 (63%) male	18/20 (90%) *IDH*-wildtype	15/18 *MGMT* promoter unmethylated	3/15 (20%) received immunotherapy before osseous metastasis discovery	9/15 (60%) received bev	Median survival after osseous metastasis discovery: 3 mo (range, 0–25)	Median overall survival: 27.6 mo (range, 6–139)

− = unmethylated; + = methylated; Bev = bevacizumab; F = female; GBM = glioblastoma; M = male; WT = wildtype.

* Therapy administered after bone metastasis was discovered.

## 4. Discussion

In this single-center case series, we identified 6 GBM patients with osseous metastases and analyzed their clinical characteristics to understand potential risk factors for GBM metastasis and the prognostic significance. Our study supports previous literature published on extracranial metastatic GBM risk factors, including male sex (5/6, [83%] of our patients) and younger age at GBM diagnosis (median 46 years old).^[4,8,10]^ Considering that young age at diagnosis is a predictor for a longer life expectancy for GBM,^[22]^ longer survival time of younger patients may allow for more time for GBM to metastasize. However, in our study, the patients had a typical life expectancy with GBM, surviving a median total of 17 months after initial diagnosis. Expanding to the cases of osseous metastases published in the literature, median overall survival was 28 months, longer than what would be typical for GBM, supporting the hypothesis that longer life expectancy may lead to higher chance of extracranial metastasis.

Four of 6 patients in our study (67%) had their primary GBM in the left temporal lobe, while 1 patient had GBM in the right temporal lobe (17%). This is consistent with a previous study of metastatic GBM showing that the temporal lobe is the most common location (52%) for primary GBM in cases with extracranial metastasis.^[4]^ Consistent with prior studies, the most common bones invaded by metastatic GBM were the thoracic vertebral bodies in 4 of 6 patients (67%).^[10]^

From a molecular perspective, prior reports of metastatic GBM may have had *IDH* mutations and today would not be classified as GBM. In the 2022 systematic review of osseous metastasis from GBM, mutational status was not known for many tumors, and 2 included cases were *IDH*-mutant.^[10]^ Under the 2021 World Health Organization classification of CNS tumors, *IDH*-mutant tumors are no longer classified as GBM. Thus, we only considered *IDH*-wildtype for our institutional analysis.

All patients in our case series had GBM, *IDH*-wildtype with unmethylated *MGMT* promoter status. Presence of *MGMT* promoter methylation is associated with increased susceptibility to alkylating chemotherapy and prolonged survival.^[23]^ Less is known about the relationship between *MGMT* promoter methylation status and extracranial GBM metastasis. However, all our patients and 83% of patients with GBM osseous metastases reported in the literature (Table 2) had unmethylated *MGMT* promoter, suggesting that a more aggressive, treatment-resistant phenotype is more common in metastatic GBM. Five of our 6 patients (83%) harbored variable alterations in tumor-suppressor gene, *TP53*, including mutations (n = 2), overexpression (n = 1), and underexpression (n = 2) (Table 1). Similarly, a study comparing the molecular features of GBM with extracranial metastases compared to GBM without extracranial metastases, showed decreased *MGMT* methylation and more frequent *TP53* mutations in patients with GBM with extracranial metastases.^[24]^

Gliosarcoma histology, which accounts for only 2% of GBM cases, is a potential risk factor for extracranial metastasis.^[9]^ In our small cohort of 6 patients, 2 had gliosarcoma (33%), further supporting this association with extracranial GBM metastasis. *EGFR* mutation is less common in gliosarcoma compared to GBM^[9]^ and neither of our patients with gliosarcoma harbored *EGFR* mutations in their tumors.

Most of our patients (4/6, 67%) received the anti-angiogenic agent, bevacizumab, prior to the discovery of their GBM osseous metastases for treatment-related vasogenic edema (n = 4) and/or for treatment of tumor progression (n = 2). Bevacizumab use has been correlated with early extracranial metastasis of GBM.^[5]^ Long-term bevacizumab use increases tumor invasiveness.^[25]^ One study also showed that bevacizumab induction of hypoxia correlated with earlier GBM metastasis in mouse models.^[26]^

Two of our patients received immunotherapies (PD-1 inhibitors), retifanlimab (n = 1) and pembrolizumab (n = 1), for treatment of GBM prior to discovery of their osseous metastases. An additional patient received immunotherapy in the form of a dendritic cell vaccine. PD-1 is a receptor that leads to immune system inhibition and interacts with PD-L1, which is expressed on GBM cells.^[27]^ Although promising, PD-1 inhibitors have not yet been shown to improve survival in GBM patients.^[27]^ An increased risk of GBM metastasis in the setting of anti-PD-1 immune checkpoint inhibitors or vaccine-based therapies would be unexpected, as the intention of the immunotherapy is to alert the host immune system to invading GBM cells, especially once they escape the blood-brain barrier. Future studies should compare the rate of extracranial GBM metastases in patients who receive immunotherapy versus those who do not to determine whether there is a paradoxical relationship between extracranial GBM and immunotherapy exposure. Of note, all our patients were lymphopenic, related to chemotherapy and radiation treatment. As they were immunosuppressed, we hypothesize PD-1 inhibitor therapy in 2 patients and the use of anti-tumor dendritic cell vaccine in 1 patient may have had less benefit.

The incidence of extracranial metastases of GBM is likely underestimated, as screening for metastatic GBM is not routinely performed.^[10]^ Although patients may experience symptoms from GBM metastases, symptoms may easily be mistaken for other complications of GBM, like steroid side effects or sequelae of the intracranial tumor.^[4]^ As life expectancy improves with treatment advancements in GBM, we may see an increase in metastatic disease, as the temporal window of opportunity for metastasis expands.

Limitations of our study include the small number of patients and the limitation of our case series to a single institution. For this reason, we referenced previously published case reports in the literature. Because osseous metastases of GBM can be asymptomatic and screening for metastatic disease is not routine, there are very likely patients who had undiagnosed metastases of GBM who were not detectable in our retrospective search and thus were not included in our study.

On the other hand, our case series bolsters the growing literature that osseous metastases of GBM not only occur but may cause symptoms. Unfortunately, osseous metastases of GBM indicate a poor prognosis, with a median survival after bone metastasis discovery of only 25 days in our cohort and 3 months in the larger group of cases in the literature (Table 2).

In conclusion, caregivers of GBM patients should be weary of the rare occurrence of extracranial metastasis, especially in patients with risk factors for such spread, such as male sex, younger age, bevacizumab use, and advanced intracranial disease. While metastatic disease does not appear to shorten GBM patients’ life expectancies, it can impact their quality of life. Future studies of the molecular mechanics of GBM mobilization outside of the brain may uncover vulnerabilities to target the malignant tumor primary headquarters in the brain.

## Author contributions

**Conceptualization:** Lauren Michelle Webb, Ugur Sener.

**Data curation:** Lauren Michelle Webb, Mason J. Webb, Ugur Sener.

**Formal analysis:** Lauren Michelle Webb, Ugur Sener.

**Project administration:** Ugur Sener.

**Supervision:** Jian L. Campian, Michael W. Ruff, Joon H. Uhm, Ugur Sener.

**Validation:** Jian L. Campian, Samantha J. Caron, Ugur Sener.

**Writing – original draft:** Lauren Michelle Webb.

**Writing – review & editing:** Mason J. Webb, Jian L. Campian, Samantha J. Caron, Michael W. Ruff, Joon H. Uhm, Ugur Sener.
